# Considerations and Challenges for Real-World Deployment of an Acoustic-Based COVID-19 Screening System

**DOI:** 10.3390/s22239530

**Published:** 2022-12-06

**Authors:** Drew Grant, Ian McLane, Valerie Rennoll, James West

**Affiliations:** Department of Electrical and Computer Engineering, Johns Hopkins University, Baltimore, MD 21218, USA

**Keywords:** COVID-19, acoustics, machine learning, respiratory diagnosis, healthcare, telemedicine, digital forensics

## Abstract

Coronavirus disease 2019 (COVID-19) has led to countless deaths and widespread global disruptions. Acoustic-based artificial intelligence (AI) tools could provide a simple, scalable, and prompt method to screen for COVID-19 using easily acquirable physiological sounds. These systems have been demonstrated previously and have shown promise but lack robust analysis of their deployment in real-world settings when faced with diverse recording equipment, noise environments, and test subjects. The primary aim of this work is to begin to understand the impacts of these real-world deployment challenges on the system performance. Using Mel-Frequency Cepstral Coefficients (MFCC) and RelAtive SpecTrAl-Perceptual Linear Prediction (RASTA-PLP) features extracted from cough, speech, and breathing sounds in a crowdsourced dataset, we present a baseline classification system that obtains an average receiver operating characteristic area under the curve (AUC-ROC) of 0.77 when discriminating between COVID-19 and non-COVID subjects. The classifier performance is then evaluated on four additional datasets, resulting in performance variations between 0.64 and 0.87 AUC-ROC, depending on the sound type. By analyzing subsets of the available recordings, it is noted that the system performance degrades with certain recording devices, noise contamination, and with symptom status. Furthermore, performance degrades when a uniform classification threshold from the training data is subsequently used across all datasets. However, the system performance is robust to confounding factors, such as gender, age group, and the presence of other respiratory conditions. Finally, when analyzing multiple speech recordings from the same subjects, the system achieves promising performance with an AUC-ROC of 0.78, though the classification does appear to be impacted by natural speech variations. Overall, the proposed system, and by extension other acoustic-based diagnostic aids in the literature, could provide comparable accuracy to rapid antigen testing but significant deployment challenges need to be understood and addressed prior to clinical use.

## 1. Introduction

Coronavirus SARS-CoV-2 and its associated disease (COVID-19) has led to unprecedented global disruptions. The rapid and uncontrolled spread of COVID-19 across the world can be largely attributed to lack of test access. A prompt, positive test allows individuals to isolate and seek treatment earlier, reducing transmission risks, disease severity, and deaths. While billions of vaccines were deployed in 2021 and 2022, experts agree that testing is critical to regulate the spread of COVID-19 [1,2] and the development of effective testing modalities that can be rapidly mobilized is crucial to ensuring pandemic preparedness in the future [3,4].

Reverse transcription polymerase chain reaction (RT-PCR) and rapid antigen testing are most often used to reduce transmissions, with rapid antigen tests offering a quicker and lower cost solution compared to RT-PCR [5]. However, rapid antigen tests (BinaxNOW by Abbott; BD Veritor by Becton Dickinson; Flowflex by ACON Laboratories; to name a few) demonstrate wide variability and significantly lower sensitivity than the gold standard RT-PCR tests; sensitivities have been reported from 44% to 79% in university screening programs [6,7]. Severe shortages of supplies and logistical challenges related to deployment have also limited testing at critical points throughout the pandemic [8]. A reliable AI-based screening tool based on easily acquirable physiological sounds (cough, speech, and breathing) would provide a simple, scalable, low-cost, and expeditious method to detect COVID-19.

The previous literature has shown that classification of COVID-19 using acoustic signatures is indeed possible: Laguarta et al. [9] achieved a 93.8% accuracy on forced-cough recordings with parallel ResNet50 deep learning architectures; Imran et al. [10] used three parallel classifier systems with a mediator to achieve a final accuracy of 92.64% (though the app predicted an inconclusive test result 38.7% of the time, which was not accounted for in the accuracy); Pahar et al. [11] applied transfer learning on a pre-trained ResNet50 architecture to achieve accuracies above 92% for cough, speech, and breathing sounds; and Pinkas et al. [12] used a three stage deep learning architecture to correctly identify 71% of positive patients. The release of public datasets, such as Coswara/DiCOVA Challenge [13,14], University of Cambridge/NeurIPS 2021 [15], and COUGHVID [16] has dramatically accelerated the development and release of new classification approaches with reported area-under-the-curve of the receiver operating curve (AUC-ROC) ranging from 0.60 to 0.95 [17,18,19,20]. Previously, the authors have also presented early work on the Coswara Dataset [21] that was the top performer in the breathing and cough tracks of the Second DiCOVA Challenge, achieving an AUC-ROC of 0.87 and 0.82, respectively [22].

Motivated by these preliminary studies, several systems have been deployed by researchers and corporate entities for public or clinical use, and a handful have applied for regulatory approval. The COVID Voice Detector by Carnegie Mellon was built on foundations of earlier voice-profiling work for vocal fold pathologies [23,24]. The system briefly went live on 30 March 2020 to offer COVID classifications, but was quickly withdrawn by the researchers due to concerns regarding data quality, clinical validity, and risk [25]. ResApp announced promising preliminary results from COVID-19 detection with cough sounds in a pilot clinical trial, based originally on pneumonia work, reportedly achieving 92% sensitivity and 80% specificity. However, an independent study of ResApp’s algorithm when deployed revealed significantly lower sensitivity (84%) and specificity of (58%), citing challenges in generalizability and training datasets [26].

Regrettably, these systems failed to address several key considerations and compounding challenges related to mass deployment, including but not limited to: (1) variability between recording equipment (computer, iPhone, Android phone, etc.), (2) model generalizability, (3) analysis of training dataset biases and statistics, (4) performance when presented with other respiratory diseases and conditions, (5) performance in the presence of additive noise, and (6) accuracy for repeated testing of a single individual. This non-exhaustive list of challenges is only partially addressed in the existing literature related to acoustic-based COVID-19 classification. Khanzada et al. [27,28] acknowledge these shortcomings, but do not provide analyses to address them. Only [29] offers an analysis regarding dataset preparation ((3) above), model overfitting ((2) above), and comparison with other respiratory diseases like asthma and bronchitis ((4) above). To the best of our knowledge, no researchers have robustly quantified system performance with environmental noise corruption, the same speaker with different classes, the same speaker with the same class, and variability between recording equipment.

To successfully deploy an acoustic-based COVID-19 classifier, it is critical to understand how these challenges will impact the detection accuracy, necessary training data, and system limitations. Therefore, this study is not centered around comparing model architectures or maximizing accuracy; instead, a specific detection system is presented as a baseline and the effects of the aforementioned challenges are quantified to understand the robustness considerations needed to deploy such a system at-large. The aim of this work is to provide a preliminary framework and understanding to other researchers on techniques to measure system robustness. While this study primarily focuses on acoustically detecting COVID-19, the concepts and principles presented can be applied to detect other respiratory diseases and serve as a useful guide for developing robust acoustic-based systems.

## 2. Methods

**Figure 1:**
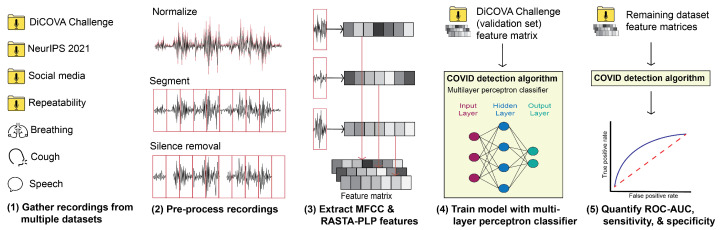
The proposed COVID-19 detection system pipeline, consisting of (**1**) data collection, (**2**) pre-processing, (**3**) feature extraction, (**4**) classification, and (**5**) performance evaluation stages.

The COVID-19 classification system, which is visually summarized in Figure 1, is developed and tested by processing cough, speech, and breathing recordings gathered from a single, large dataset. The baseline classification schema follows traditional and standard audio machine learning systems [30] and is tested on multiple datasets and conditions to understand its overall performance.

### 2.1. Datasets

**Figure 2:**
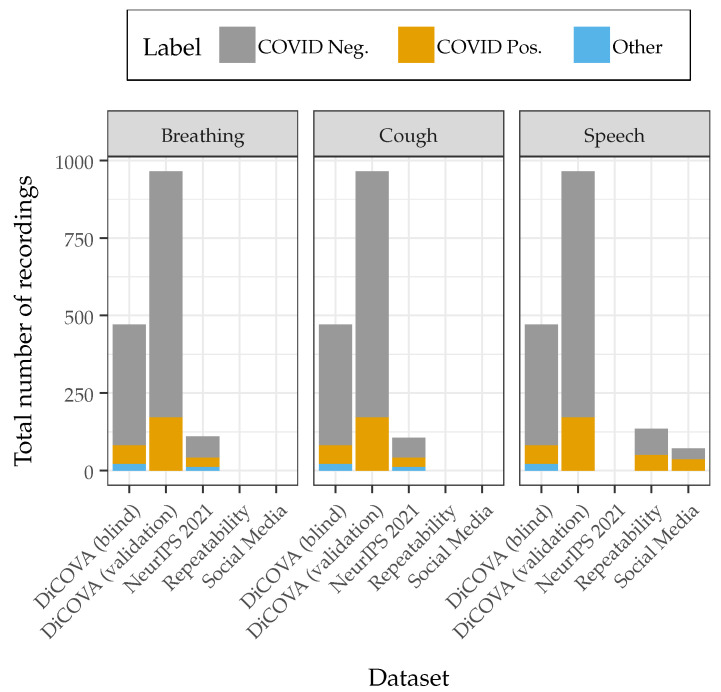
The number of recordings included in each of the five datasets that were used to train and test the COVID-19 detection system. The DiCOVA Blind and NeurIPS 2021 Datasets include subjects with other self-reported respiratory conditions. All recordings were from unique subjects, except for the Repeatability Dataset which included ten replicate recordings from each subject.

Four data sources were used to train and test the COVID-19 classification system. The model is trained using the development subset of the Second DiCOVA Challenge Dataset [22] and then tested across four additional datasets. A summary of the datasets and the included physiological sound types is presented in Figure 2.

#### 2.1.1. DiCOVA Validation and Blind Datasets

The Second DiCOVA Challenge Dataset includes crowdsourced sound recordings derived from the Coswara Dataset [22]. The challenge consisted of two data cohorts: a ‘validation’ set used for model training and cross validation, and a ‘blind’ set for blind testing and evaluation. Both datasets include ‘heavy cough’, ‘deep breathing’, and ‘normal counting’ recordings for each subject, which were voluntarily submitted via web application along with qualitative information on age, gender, health status, symptoms, pre-existing respiratory ailments, and comorbidities. The validation dataset consists of 965 subjects, of which 172 (17.8%) self-reported as being COVID-positive. The blind test set consists of 471 subjects, of which 71 (15.1%) self-reported as being COVID-positive. The DiCOVA datasets were used for initial training and baseline performance characterization across the three sound types.

#### 2.1.2. NeurIPS 2021 Dataset

The NeurIPS 2021 Dataset includes crowdsourced cough and breathing sound recordings collected by the University of Cambridge and released for scientific exploration [31]. The recordings were voluntarily submitted via a web- or Android-based application with information on symptoms, asthma diagnosis, and COVID status. The cough subset contains 106 subjects; 31 (29.3%) self-reported with COVID-19, 11 (10.4%) self-reported with asthma, and the remaining 64 (60.4%) self-reported as healthy. The breathing subset contains 99 subjects; 68 (68.7%) self-reported with COVID-19, 11 (11.1%) self-reported with asthma, and the remaining 31 (31.3%) self-reported as healthy. This dataset was used to evaluate the generalizability of the COVID-19 classification system to unseen data.

#### 2.1.3. Social Media Dataset

The Social Media Dataset was compiled by gathering speech recordings from individuals available online through television, video sharing platforms, and social media. The standard procedure to collect recordings was as follows: several news (e.g., New York Times, CNN, Fox News) and social media sites (e.g., Twitter, TikTok, Facebook, Instagram, YouTube) were searched with standard strings for recordings of subjects who had self-reported as testing positive for COVID-19 within the last seven days. Recordings of the same subject were then gathered that were taken at least one month prior to the positive COVID-19 report to ensure no overlap with incubation or asymptomatic periods. Media were included or excluded following precise criteria related to length, noise, and information available; the inclusion and exclusion criteria can be found in the collection protocol at the link below. The final dataset includes 36 subjects, each with a negative and positive COVID-19 recording. The data, along with detailed descriptions of the media, including the subjects, COVID-19 status, site used, link to media, search strings used, and quality assessment can be found at https://github.com/drewgrant/COVIDAudioSocialMediaDataset (accessed on 11 July 2022).

The dataset was gathered to understand the system’s generalizability and ability to detect changes in a single individual’s COVID-19 status. The limitation of this dataset is that subjects speak in an unstructured, unscripted, and natural manner. This poses challenges compared to the structured speech of the DiCOVA Validation Dataset, in which participants count to twenty normally. Nonetheless, the dataset is included as a meaningful contribution to analyze the robustness of the system.

#### 2.1.4. Repeatability Dataset

The Repeatability Dataset was compiled by gathering speech recordings of participants in a similar fashion to the Coswara/DiCOVA Dataset process. Eleven participants (6 COVID-negative participants, 5 COVID-positive participants) followed the standard DiCOVA Dataset process for speech, counting normally from one to ten, and voluntarily submitted recordings via web application. The participants repeated this process ten times, moving locations within their home between each recording. The subjects also provided qualitative information (age, gender, health status, symptoms, pre-existing respiratory ailments, and comorbidities) following the standard set of questions from the Coswara Dataset. This dataset was used to evaluate the generalizability of the COVID-19 classification to unseen data and the replicability of the system when used repeatedly by a single speaker.

### 2.2. Classification Schema

The baseline classification system extracts RelAtive SpecTrAl-Perceptual Linear Prediction (RASTA-PLP) and Mel Frequency Cepstral Coefficient (MFCC) features and performs classification via multilayer perceptron [21]. This schema was the highest performing system in the Second DiCOVA Challenge [22]. The system reported here has been slightly modified for standardization across sound events; multilayer perceptron and 25 MFCC and MFCC-Δ features are used across all sound events, but RASTA-PLPs of model order 25, 20, and 22 were used for speech, breathing, and cough analysis, respectively, determined by empirically from the previous work. The classification method involves three main stages: preprocessing, feature extraction, and classification.

#### 2.2.1. Pre-Processing

All audio recordings had a sampling rate of 44.1 kHz. Recordings were normalized to an amplitude range within ±1, and segmented into 40 ms windows with 50% overlap. Long time windows were used here to emphasize harmonics, which have been previously analyzed to detect hoarseness [32], a common symptom of respiratory illnesses. The short term energy of the windows was thresholded to perform sound activity detection (SAD) and remove silent segments from each recording [30,33]. Windows with energy below the threshold of 0.0001 were considered to be silence and removed. This threshold was determined empirically to balance system performance across all three sound types in recordings without added noise. Due to the nature of the recordings and the sound activity detection thresholding, the total number of windows for each subject varied with the recording and voiced audio lengths.

#### 2.2.2. Feature Extraction

The system uses MFCC and RASTA-PLP acoustic features, which are widely found in speech and sound analysis. MFCCs logarithmically warp audio signals in Mel-scale filter banks to emulate human perception [34]. MFCCs are extracted by applying a Mel-filter bank to the short-time power spectrum of a signal, taking the logarithm, applying the Discrete Cosine Transform, and ‘liftering’ (or, multiplying the whole cepstrum by a rectangular window centred on lower quefrencies) to discard the higher cepstral coefficients and retain the number of desired coefficients [30,35]. MFCCs encode high-level spectral content: the lower-order coefficients describe the overall spectral shape of the signal, while the higher-order coefficients represent finer spectral details, such as pitch and tonal information [36]. MFCCs have been used in countless state-of-the-art acoustic systems [37,38,39,40]. The changes and trajectories of the MFCCs (commonly referred to as ‘MFCC-Δs’) capture spectral variations and dynamics. MFCC-Δ coefficients are computed by taking the first derivative of the MFCCs with respect to frames. The COVID-19 classification system extracts 25 MFCC and 25 MFCC-Δ features.

RASTA-PLP features bandpass filter and nonlinearly compress audio signals to reduce the effects of additive noise and channel effects [41]. RASTA filtering applies a band-pass filter to each frequency sub-band to smooth over short-term noise variations and remove channel distortions in the signal [30]. PLP is a feature representation acquired via psychoacoustic transformations in critical band analysis, equal loudness, pre-emphasis, and intensity-loudness prior to employing the linear prediction algorithm [42] on windowed speech [30]. Combining the RASTA and PLP algorithms generates a more robust feature representation that reduces the effects of diverse recording equipment, speaker-dependent information, and the wide range of acoustic environments that one might find in crowdsourced datasets. To the best of the authors’ knowledge, this was the first system to pair MFCC and RASTA-PLP features for COVID detection when it was originally presented [21]. RASTA-PLPs of model order 25, 20, and 22 were determined empirically to optimize system performance and used for speech, breathing, and cough analysis, respectively.

**Figure 3:**
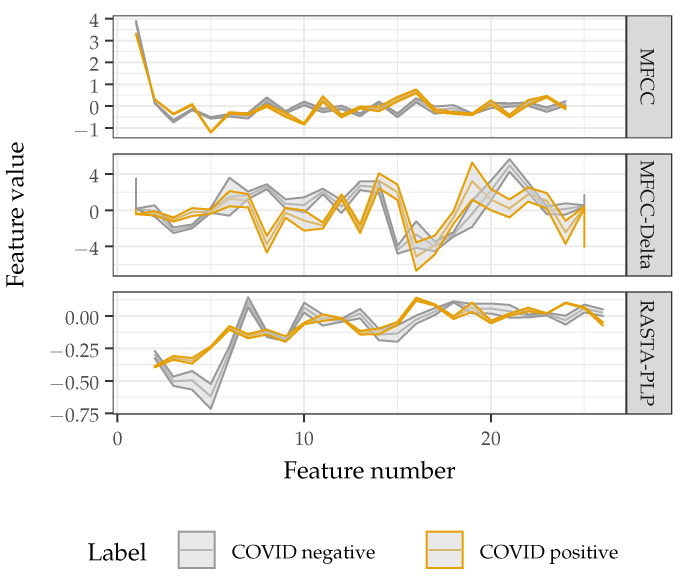
Average and standard deviation of the feature values extracted from three frames of a single subject saying ‘bye’ both with and without COVID.

Figure 3 shows the average and standard deviation of the feature vectors extracted from a single subject (subject nine from the Social Media Dataset) saying ‘bye’ in two recordings: (1) without and (2) with COVID. These frames were chosen because the classifier correctly identified the presence or absence of COVID across all frames. The figure demonstrates the subtle differences that occur between features in the two COVID cases, even for a single speaker saying the same syllable. These subtle differences in the feature vectors are what drive the need for advanced machine learning techniques for classification.

#### 2.2.3. Classifier Description

The MFCC, MFCC-Δ, and RASTA-PLP features for each signal frame are concatenated to create a single feature vector for each frame of a subject’s recording. The classification model processes the feature vectors and returns a probability score for each frame. The probability scores are averaged across all frames of the recording and an optimal classification threshold derived from receiver operating characteristic (ROC) analysis is applied to determine a probable class: COVID positive or COVID negative.

A multilayer perceptron classifier was used due to its ability to effectively model complex and nonlinear relationships and its ease of implementation [43]. The multilayer perceptron classifier was implemented using scikit-learn 1.1.3 toolkit [44] with the following empirically selected parameters: Limited-memory Broyden-Fletcher-Goldfarb-Shanno (lbfgs) solver, 0.000001 alpha, 1000 maximum iterations, 3 hidden layers with [100, 300, 100] neurons, and all other parameters set to the default. The previous work [21] explored classification model comparison primarily between multilayer perceptron and random forest, a popular ensemble-based classification algorithm. Other deep learning algorithms were not considered because of their black-box nature; challenges with repeatability and replicability from hyperparameter selection, initialization states, random seeding, and data selection; and limited training data [45].

#### 2.2.4. Performance Evaluation

To measure the performance of the classifier in various discrimination and robustness tasks, the sensitivity, specificity, and AUC-ROC were computed [46,47]. These metrics are commonly used for evaluating binary classification tasks [48]; traditional performance metrics, such as overall accuracy or error rate, are severely limited when handling imbalanced data [49]. Sensitivity and specificity describe the accuracy of binary diagnostic tests for both classes at a specific decision threshold by indicating the rate of true positives and false positives, respectively.

ROC curves relate the sensitivity and specificity across all possible decision thresholds. As the classifier improves, the ROC curve becomes steeper and increases the AUC-ROC, a metric that provides a generalized, threshold-independent understanding of the classifier’s performance. While other metrics could be used (i.e., precision, recall, F1 score, etc.), AUC-ROC is common for diagnostic tests and data science applications [50,51], including COVID detection tasks [9,10,22], and allows for performance standardization and model comparisons across datasets and studies.

The ROC curve informs the selection of a decision threshold value for deploying the classification system; average probability scores above the threshold classify the subject as having COVID. The threshold value is typically selected to balance the sensitivity and specificity for the use-case of the classifier, since it can be difficult to agree at which threshold it is acceptable to risk missing disease. In this work, the decision threshold is selected as the point that maximizes Youden’s J Statistic [52]. For the DiCOVA Validation Dataset, a single development threshold is used throughout the paper, referred to as the ‘development threshold’. The remaining datasets are evaluated both with this development threshold and with an ‘optimal threshold’ that optimizes Youden’s J Statistic for that specific dataset.

Five-fold cross validation was used for model training and validation within the DiCOVA Validation Dataset, as specified in the Second DiCOVA Challenge [22]. The DiCOVA Validation Dataset results are the average AUC-ROC across all folds, as well as the average sensitivity and specificity for an optimal decision threshold across all five folds. The performance metrics obtained using an earlier version of the proposed system with the DiCOVA Blind Dataset were validated externally by the DiCOVA team (presented in [22] as T-14), making these results highly credible and objective.

### 2.3. Real-World Deployability Testing

To evaluate the robustness of the system for challenges faced in real-world deployment, the baseline performance is reported as a benchmark to understand how varied recording equipment, subject groups, and background noise will impact the system performance via statistical testing and comparison of AUC-ROC, sensitivity, and specificity values.

#### 2.3.1. Recording Device

Of the datasets analyzed, only NeurIPS provided information on the device, either web- (36%) or Android-based (64%), used to capture the recordings. The AUC-ROC of the system with recordings solely from web-based or Android-based devices are compared to understand if the recording device impacts the system performance.

#### 2.3.2. Model Generalizability

The AUC-ROC, sensitivity, and specificity are obtained from testing the system on the DiCOVA Validation, DiCOVA Blind, NeurIPS 2021, Social Media, and Repeatability Datasets to understand the (1) generalizability of the model, (2) if any overfitting occurred in the initial training, and (3) how widely applicable the model and the development threshold is when applied to unseen, uncorrelated data. ROC curves, AUC-ROC values, and sensitivity and specificity values at the development and optimal thresholds are compared.

The NeurIPS and Repeatability Datasets are used to test on data that is ostensibly collected in a similar fashion but could diverge in the latent distribution from the initial DiCOVA Validation Dataset due to population, instructions, or recording equipment. The Social Media Dataset is also used to further challenge the model by using unstructured audio with natural, conversational speech.

#### 2.3.3. Model Complexity

To rapidly scale a classification system for wide-scale deployment and assess recordings in an efficient manner, a model that balances high classification performance and low complexity is preferred. The feature dimensions, classification models, and AUC-ROC for the system proposed here and for others found in the literature for cough sounds are compared.

#### 2.3.4. Confounding Factors

The demographic diversity of subjects that provided recordings for the datasets used in this study could impact their sound production and the system performance. The percentages of recordings from subjects with different genders, age groups, symptoms, and other respiratory conditions are shown in Table 1. The majority of subjects were male and 15 to 45 years old. Only a small percentage of subjects (∼29%) reported whether they were asymptomatic or symptomatic and a small percentage (∼30%) reported whether or not they had other respiratory conditions.

To understand how the system performance changes due to these confounding factors, statistical analyses were performed using the average probability scores from all subjects and datasets. Due to the non-normal distribution of the average probability scores (*p*-value <0.001 via Shapiro–Wilk test), non-parameteric analyses of variance (Wilcoxon or Kruskal–Wallis tests) were used to determine if gender, age group, symptoms, or other respiratory conditions had a significant effect on the probability score distributions when grouped by the sound type and COVID status. Recordings without a gender or age label were removed from the analysis and the significance level was set to 0.05.

In addition to the statistical analyses, the baseline AUC-ROC was compared to the AUC-ROC on data subsets by gender (all male or all female), age (15–29, 30–45, 46–59, or over 59), other respiratory diseases (yes or no), or symptom status (asymptomatic or symptomatic). Though these are not exhaustive analyses of model bias, the testing provides valuable insight into potential performance differences based on the confounding factors that would need to be considered prior to deploying acoustic-based systems more widely.

#### 2.3.5. Additive Noise Injection

To test the robustness of the system against the presence of ambient noise, the baseline classification system was tested on varying levels of both stationary and nonstationary noise. Recordings from the DiCOVA Validation and Blind Datasets were artificially corrupted with ambient noise and other artifacts. Noise segments were randomly chosen from a noise database and added to the clean signals at prescribed signal-to-noise (SNR) levels. This technique ensures the ability to directly compare performance of the various loudness conditions, but also allows for some randomization in the type of ambient sounds being added.

The fourteen noise types originally used in [53] are included here: air conditioner, announcements, appliances (washer/dryer), car noise, copy machine, door shutting, eating (munching), multi-talker babble, neighbor speaking, squeaky chair, traffic, road, typing, vacuum cleaner, speakers reading from passages. White, pink and Brownian noise were also included. Sounds were sorted following the technique outlined in [54]: the average Power Spectral Entropy value for each recording was used as a stationarity index such that minimum entropy occurs for highly variable sounds (nonstationary noises) and maximum entropy occurs when the spectral distribution is uniform (stationary noises).

Ambient noises and recordings were mixed at seven different equally spaced SNR levels from 0 dB (extremely noisy) to 60 dB (quiet). All the final mixed files are normalized to −25 dBFS (decibels relative to full scale of the digital waveform). A combination of three training conditions and three testing conditions were created for each SNR level. The system was trained on one of three training sets: clean (unprocessed) dataset, the dataset with added stationary noise, or the dataset with added nonstationary noise. Each of these systems was then tested on one of three testing sets, which were generated following a similar process: clean (unprocessed) dataset, added stationary noise, and added nonstationary noise. This process was repeated for each of the sound types (speech, breathing, cough). The AUC-ROC of the classifier for each of the noise levels (0 to 60 dB) and nine train-test pairs is then calculated for comparison.

#### 2.3.6. Single Speaker Repeatability and Discrimination

A longstanding fundamental challenge in speech processing is repeatability [34]; intra-speaker variability and natural variations in speaking rate are unavoidable and that cause no two utterances to be the exact same [55,56]. Two cases are considered to measure the performance of the classifier when used repeatedly by participants: whether the model (1) performs consistently when presented with many recordings from a single participant with the same status, and (2) correctly identifies when a single participant’ status changes.

For the first test, the baseline model is tested on ten recordings from each participant in the Repeatability Dataset. The number of correct and consistent classifications when using the development and optimal thresholds are compared. Probability scores for each frame of speech across the ten recordings of a subject are also analyzed using the Kruskal–Wallis non-paramateric test to assess intra-speaker differences.

For the second test, the baseline model is tested on paired recordings of a single speaker with and without COVID from the Social Media Dataset. The number of subjects with correct classifications for both COVID states are determined. Subjects that were misclassified for one or both COVID states are grouped by whether the misclassification occurred due to the chosen detection threshold or if the system reversed the actual COVID states for the subject. The probability scores from each frame for all subjects with and without COVID are also assessed with the Wilcoxon test to determine if a significant difference exists for a single subject with and without COVID.

## 3. Results and Discussion

The acoustic detection system classified subjects with and without COVID-19 via breathing, cough, and speech sounds with an average AUC-ROC of 0.77. The real-world deployability testing procedures highlight several challenges, including diverse recording devices, training and testing data mismatches, noise corruption, and natural variations in speaking, that could cause the system performance to decrease in real-world settings, but also highlight the system’s robustness to confounding factors.

### 3.1. Recording Device

**Figure 4:**
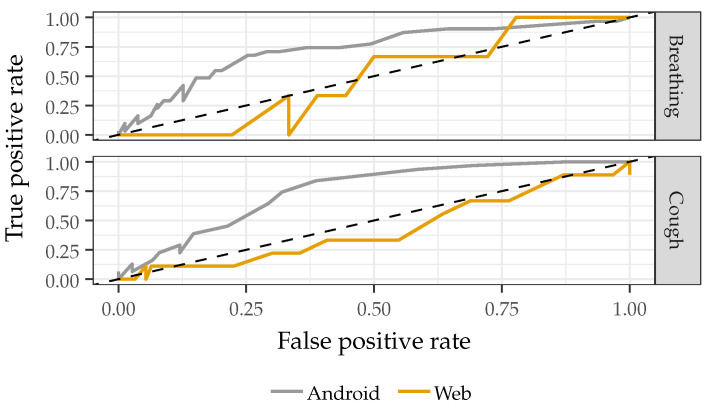
ROC curves comparing the system’s performance using recordings from Android and web-based devices. The system displays decreased performance with recordings from web-based devices.

As shown in Figure 4, the classifier shows significantly decreased performance with web-based recordings (AUC-ROC 0.48 for breathing and 0.42 for cough) compared to Android recordings (AUC-ROC 0.73 for breathing and 0.75 for cough). Possible explanations for this performance degradation could include that the subject is less likely to speak directly into the microphone using a web-based device or there is greater variability in the soundcard pre-processing for web-based devices, but additional testing data with varied recording devices is required to determine why specific devices would degrade classifier performance. Due to the decreased performance of the classifier with web-based recordings, only Android-based recordings from the NeurIPS Dataset were included for subsequent analyses when the NeurIPS data is used. It is important to note that only the NeurIPS Dataset provided information on the recording devices used, so it is unclear how varying recording devices impacted the performance across all datasets.

### 3.2. Model Generalizability

**Figure 5:**
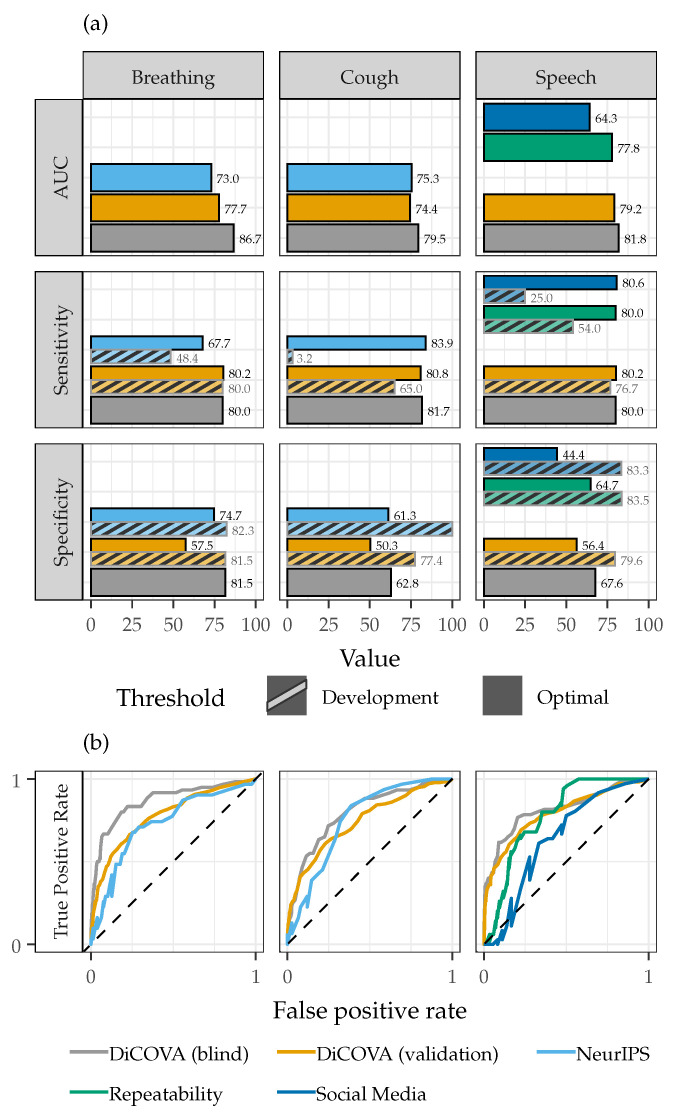
System (**a**) AUC-ROC, sensitivity, and specificity values and (**b**) ROC curves for breathing, cough, and speech sounds from all datasets classified individually.

Figure 5 shows the AUC-ROC, sensitivity, and specificity values along with the ROC curves for each dataset classified individually. The average, minimum, and maximum AUC-ROC values across all datasets and sound types is 0.77, 0.64, and 0.87, which indicates that the system accurately identifies subjects with COVID using breathing, cough, and speech sounds from various datasets that differed from the initial system development set. Across the AUC-ROC values of breathing, cough, and speech sounds, the classifier performed best with the DiCOVA Blind Dataset, which was expected as this dataset is the most similar to the DiCOVA Validation Dataset used for system development. The lowest AUC-ROC (0.64) was obtained using the Social Media Dataset. The performance degradation for this specific dataset was also expected as the recordings contained spontaneous speech, rather than the structured speech contained in the DiCOVA Validation Dataset used for the system development. To the best of the authors’ knowledge, no other acoustic respiratory disease system has been tested on spontaneous conversational speech, yet the performance is not considerably worse than the system presented here with structured speech datasets (DiCOVA, Repeatability) or other systems that are trained and tested with fixed speech phrases [57,58].

In Figure 5, the sensitivity and specificity values were calculated using both the development and optimal thresholds. Using the optimal threshold, which differs across each dataset and sound type, the system demonstrated an average sensitivity and specificity of 79.5% and 62.1%, respectively. However, when the system is deployed in a real-world setting, the correct classification of a subject is unknown, such that an optimal threshold cannot be calculated. Instead, the threshold must be determined from the available system training data. Using the development threshold, the system was less accurate at identifying subjects with COVID, as indicated by a decreased average sensitivity of 50.3%.

**Figure 6:**
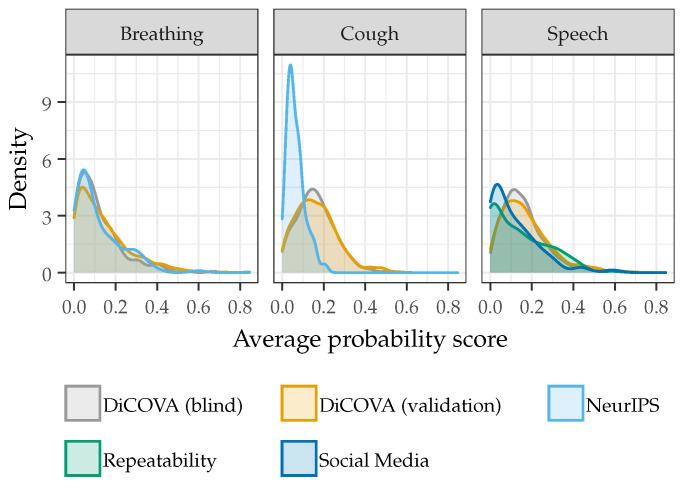
The distribution of average probability scores for all recordings in a particular dataset.

The changes in performance with the set development threshold can be understood by considering the distributions of probability scores across each dataset, shown in Figure 6. For breathing sounds, the average probability score distributions across the DiCOVA Blind, DiCOVA Validation, and NeurIPS Datasets are comparable. As such, the sensitivity and specificity values for the optimal and development thresholds are typically comparable. However, for cough and speech sounds from the NeurIPS, Repeatability, and Social Media Datasets, the average probability score distributions are considerably different than the DiCOVA Datasets. This leads to considerable variation in the measured sensitivities and specificities across these datasets when using the development and optimal thresholds. To overcome this performance degradation when a specific classification threshold must be chosen, the training dataset must include more recordings that are representative of those found across all datasets or a strict protocol should be implemented to guarantee that the training and testing recordings are collected in a similar manner.

Stowell et al. noted similar difficulties with mismatches between training and testing conditions when using deep learning methods to acoustically detect bird calls with various noise levels, low SNRs, and wide variations in bird call types [59]. Stowell et al. emphasized that automatic detection results should be treated with caution because true generalization remains difficult given the mismatch in training and testing conditions. The best solution is to obtain training data that closely match the conditions of the testing data.

### 3.3. Model Complexity

Table 2 shows a comparison of model complexity (low, moderate, high) and feature dimensionality from other systems in the literature, demonstrating the proposed system’s competitive diagnostic performance with lower dimensionality. Systems with high model complexity are more susceptible to overfitting and require devices with extensive computational resources. The proposed system’s low model complexity allows for model optimization, scaling, rapid testing of new unseen data, and deployment on low-resource devices.

### 3.4. Confounding Factors

To assess the system robustness to possible confounding factors, such as (1) gender, (2) age, (3) the presence of symptoms, or (4) other respiratory diseases, statistical analyses were performed using Wilcoxon and Kruskal–Wallis tests on the average probability scores returned by the classifier for each recording. Significant differences (*p* < 0.0001) between the average probability scores of subjects with and without COVID across sound types were identified, confirming separability between groups. Due to this difference, subsequent analyses were grouped by COVID status and sound type. This resulted in six different groupings for each confounding factor analyzed: COVID positive and negative within each sound class of breathing, cough, or speech. Significant differences were identified for the average probability scores of males and females without COVID for breathing (*p* = 0.02), cough (*p* < 0.0001), and speech (*p* = 0.002); however, no significant differences were identified between males and females with COVID across sound types. A significant difference (*p* = 0.015) was also identified between cough recordings from subjects without COVID with other respiratory conditions and from subjects without COVID with no condition listed. No other significant differences (*p* > 0.05) between age groups, symptom presence, or other respiratory conditions were identified across subjects with and without COVID for cough, speech, and breathing.

These results indicate that the probability score from the classifier for COVID-positive patients is not being influenced by confounding factors, including gender, age, and the presence of other respiratory conditions or symptoms. However, these factors seem to influence the probability score for COVID negative patients under certain conditions, as indicated in the analysis above.

**Figure 7:**
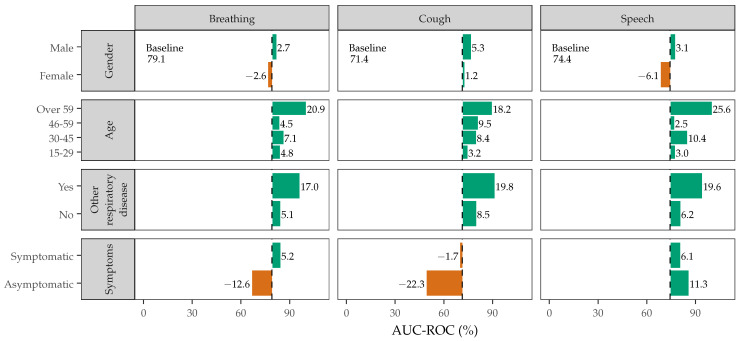
The AUC-ROC of the system when tested on various subsets of data according to gender, age, symptom reporting, or other respiratory disease and compared to the system performance with all datasets of a specific sound type combined (baseline). Green or red bars indicate an increase or decrease above the average performance, respectively.

To support these statistical analyses, Figure 7 shows the system AUC-ROC when the trained models are only tested on specific subsets of the data to identify performance gaps due to gender, other respiratory diseases, and symptoms. While the analysis is limited due to the the small percentage of subjects who reported symptoms or other respiratory conditions, as shown in Table 1, the results do point out areas the require further consideration and additional data. When comparing system performance with gender, the system shows a greater than 5% decrease in performance with females. Since the DiCOVA Validation Dataset used for model training is overwhelmingly male (72.5% male vs. 27.5% female), one would expect biasing towards male subjects and that more females in the training data might increase the performance with female subjects. All datasets except the NeurIPS 2021 contained gender labels.

The largest changes in system performance (up to 25%) occur when assessing subjects based on age. It is important to note that only the DiCOVA Blind and Repeatability Datasets contained age labels. The results suggest that the system is more likely to accurately detect whether subjects over the age of 59 are COVID positive or negative. This may imply that elderly subjects undergo significant vocal changes when infected with COVID, which may be expected considering older adults have a greater risk of becoming severely ill from COVID and requiring hospitalization.

The second largest changes in system performance (up to 22%) occur when assessing subjects with other respiratory conditions or asymptomatic COVID. The difference in performance when a subject reports ‘yes’ to other respiratory disease suggests that the system is more likely to accurately detect whether or not subjects with other respiratory diseases are COVID positive or negative. This may suggest that subjects with other respiratory diseases have acoustic features that overlap with features that the system uses for COVID detection. However, it should be noted that majority of the subjects with ‘other respiratory diseases’ were from DiCOVA Blind Dataset, which the system performed the best on. This is important to note because the DiCOVA Blind Dataset most closely matches the conditions of the DiCOVA Validation Dataset that was used to train the system, so it is not surprising that the system performs well on this subset. Nonetheless, the system’s impressive performance on subjects with other respiratory diseases is particularly noteworthy as a study by Mouliou et al. reports that clinical diagnostics and laboratory test are affected not only by pre-existing diseases but also the total health status of the subject [63]. Specifically, diseases ranging from asthma, COPD, and pneumonia have been shown to impact the performance of PCR tests and cause false negatives.

For symptom reporting, the results indicate that the system performs better using speech to detect asymptomatic COVID-positive subjects compared to breathing and cough. This may suggest the system is able to detect subtle nuances of asymptomatic COVID-positive subjects that are only perceptible through the dynamic variations of phonemes produced in speech. Conversely, fewer asymptomatic subjects are accurately classified using cough sounds, which is particularly interesting considering many of the acoustic COVID detection systems proposed by researchers use cough sounds.

### 3.5. Additive Noise Injection

**Figure 8:**
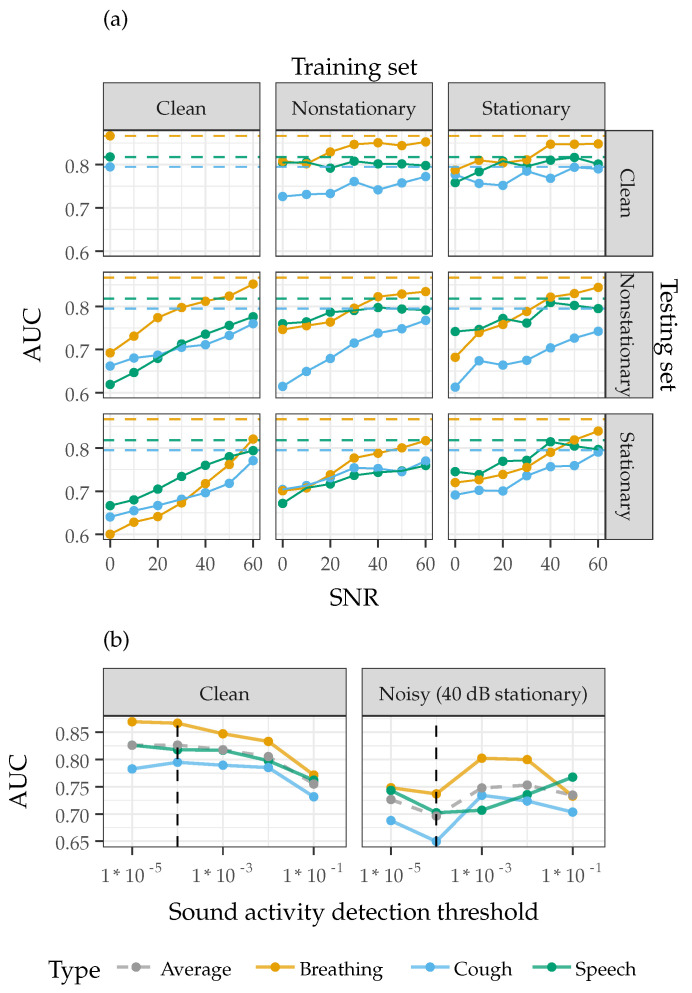
(**a**) System performance when injecting stationary and nonstationary noise of varying SNR levels into the train-test data for breathing, cough, and speech recordings. (**b**) The measured AUC-ROC at various SAD thresholds for the baseline classification system using recordings that are clean and with added stationary noise at a signal-to-noise (SNR) level of 40 dB. The chosen sound activity detection threshold (indicated by the dashed line) optimized the average performance across breathing, cough, and speech sounds for clean recordings.

Figure 8a shows the AUC-ROC of the system when trained and tested with stationary and nonstationary noise at sound levels from 0 dB to 60 dB. Added noise of any level or type decreases the performance of the system, with an overall linear trend between added noise level and system performance. Cough, speech, and breathing sounds typically exhibit similar trends between system performance and added noise level, except for cough sounds, which are particularly susceptible to training on recordings with nonstationary or stationary noise and testing on recordings with nonstationary noise. It is clear that the presence of noise in real-world deployment would significantly decrease the performance of the system when trained on clean, well-controlled recordings. Overall, when noisy conditions are expected, the results indicate that breathing and speech are less susceptible to added noise, likely due to their more consistent spectral profile compared to speech. Inclusion of noisy and non-ideal recordings in the training process has the potential to increase robustness of the system, especially in very noisy conditions. However, as noise in the training set increases, the classifier seems to incorrectly attribute certain features to the detection task, as shown in the case when tested on only clean data after being trained on noisy data. A balance is needed between inclusion of noise in the training dataset and the control of noise in the end-use.

To improve the system performance with added noise, a different SAD threshold, which determines what frames are considered sound or noise, could also be used, as shown in Figure 8b. A SAD threshold of 0.0001 was used throughout the paper to measure system performance, which was found to optimize the average performance across breathing, cough, and speech sounds in the no-noise condition. When classifying recordings with 40 dB of added stationary noise, a higher SAD threshold is found to increase the system AUC-ROC since a lower threshold introduces more noise frames in the analysis. Meanwhile, in the quiet cases, SAD algorithms with thresholds set too low fail to remove any silence or noise while SAD algorithms with thresholds set too high can be overly aggressive and remove key information. Advanced SAD algorithms that dynamically adjust the threshold or calculate optimal thresholds based on calibration recordings may be deployed in the real-world scenarios. However, if noise is sufficiently controlled in the end-use, SAD algorithms with reasonable fixed thresholds would work sufficiently, and are in-fact preferable when the levels of noise and speech are not known in advance [64].

### 3.6. Single Speaker Repeatability and Discrimination

**Figure 9:**
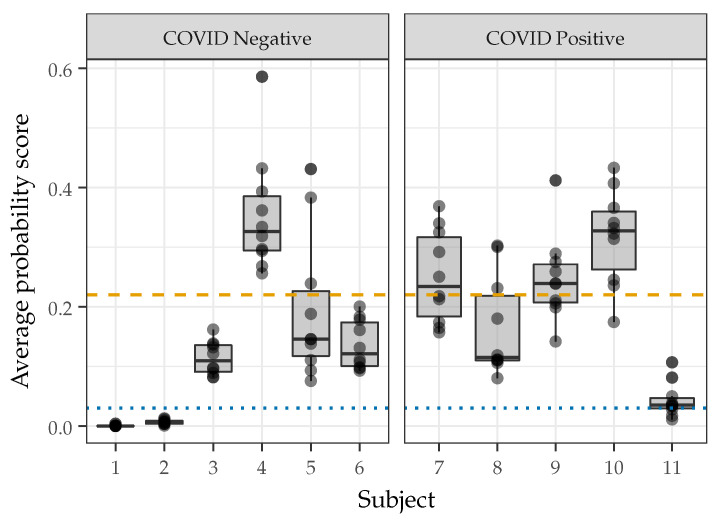
Average probability scores measured from eleven subjects counting in ten separate recordings. The blue dotted line indicates the optimal classification threshold and yellow dashed line indicates the development threshold.

Figure 9 shows the average probability scores across each of the ten recordings for subjects included in the Repeatability Dataset. The blue dotted line indicates the optimal threshold for this specific dataset (0.03) which was computed using Youden J’s Statistic for all the recordings, while the yellow dashed line indicates the development threshold (0.22). A recording with an average probability score above the threshold is classified as a subject with COVID. The results suggest that a single subject would not have a consistent COVID classification based on the chosen probability threshold. The average probability score determined by the system from the same speaker can be variable across different times or locations or with variations in the speech production.

Visually, it may appear that the optimal threshold results in poorer classification results than the development threshold in Figure 9; however, the optimal threshold results in only four false negative recordings compared to 27 false negative recordings using the development threshold. Additionally, when the optimal threshold is used, 64% of the recordings are correctly classified. Six subjects are correctly classified across all recordings, four subjects are misclassified across all recordings, and one subject has varying classifications across the ten recordings. In comparison, the development threshold correctly classifies 61% of recordings. Five subjects are correctly classified across all recordings, one subject is misclassified across all recordings, and five subjects have varying classifications across the ten recordings. Statistical testing revealed that all subjects had significant differences (*p* < 0.002) between the average probability scores measured from each frame across their ten recordings. This indicates that natural variations in speaking patterns or slight changes in the recording environment leads to significant differences in the measured probability score distributions.

**Figure 10:**
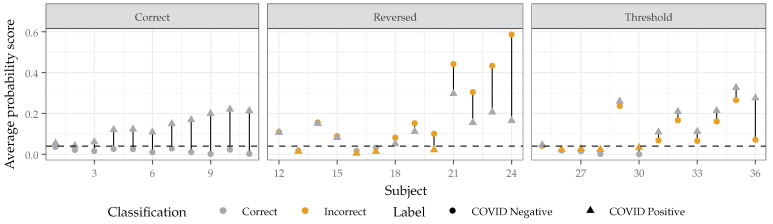
The predicted probability scores for subjects in the Social Media Dataset whose non-COVID and COVID recordings were tested on the system. The predicted probability scores illustrate that the system is able to detect differences in the subject’s non-COVID and COVID recordings; however, the threshold value limits the system’s ability to make accurate classifications.

In the Social Media Dataset, similar or greater variability is likely to be present as the recordings had greater variations in time and speech type. Figure 10 shows the average probability scores of the 36 subjects included in the Social Media Dataset. The optimal threshold for this specific dataset (0.04) was used to determine a classification; 64% of recordings were correctly classified. The development threshold correctly classified only 49% of recordings in this case, likely due to the mismatch in spontaneous and structured speech. In Figure 10, the subjects are ordered based on (1) whether the system correctly classified the recording before and while the subject had COVID (‘correct’), the probability score when the subject did not have COVID was greater than when the subject did have COVID (‘reversed’), or if a different threshold would have correctly identified both states of COVID (‘threshold’), and (2) the difference between the average probability score. Eleven subjects were correctly classified, thirteen subjects had reversed probability scores, and twelve subjects had incorrect classifications due to the chosen threshold.

Of the thirteen subjects that had reversed probability scores, nine had average probability scores that differed by less than 0.04. Of the twelve subjects that were misclassified due to the threshold, eight had average probability scores that differed by less than 0.04. This indicates that a large number of subjects were not correctly classified for both states of COVID as there was not an easily detectable change in the average probability score with and without COVID, possibly because any changes were confounded with varying speech or no changes occurred. Although the difference between average probability scores with and without COVID were frequently small, the majority of subjects did show a significant difference between the average probability scores with and without COVID, except for six subjects (13, 14, 25, 27, and 30). For subjects with average probability score differences greater than 0.04, 13 out of 18 were correctly classified, which does show promise that an individual can be correctly classified during both states of COVID when the relative changes in the average probability scores are considered.

However, it seems that separating out the changes in the average probability scores that occur due to either the variations in speaking or the COVID status remain ambiguous with the chosen system. Interestingly, taking the natural variation of physiological sounds into account (in the form of the distribution of scores in single recording) could provide additional information for classifying COVID; the system has an AUC-ROC of 0.79, 0.72, and 0.75 for breathing, cough, and speech sounds when using the standard deviation of the probability scores across frames rather than the mean probability scores.

A similar trend was reported in [65] where the authors reported the effects of different respiratory diseases on speech production. Lee at al. found that the best predictors for differentiating between speech of healthy subjects and subjects with asthma, sarcoidosis, or emphysema were the mean and standard deviations of time-related variables, which highlighted the key differences in how long healthy subjects speak uninterrupted by long pauses to breathe compared to subjects with a disease. The findings here have clear similarities that warrant further investigation in the future.

### 3.7. Summary and Limitations

The proposed acoustic-based detection system offers promising strides towards cost-effective and low-resource, rapid testing of COVID-19. Across the four data sources and three sound types included in the study, the system demonstrated an average AUC-ROC of 0.77. In general, COVID-19 positive classifications were not informed by other confounding factors, such as gender, age, and the presence of symptoms or other respiratory conditions. The simulated testing for real-world deployability highlighted several weaknesses of this system that are likely applicable to other acoustic-based COVID-19 detection systems and could degrade the system performance when moved out of a research setting, including performance variations with the recording equipment used, presence of background noise, and the natural variation of physiological sounds. It is important to note that the presented work is not intended to be a comprehensive analysis of all the issues concerning deployment of acoustic-based COVID screening systems, but rather propose framework and provide preliminary results to understand deployment challenges that need to be addressed to allow for reliable acoustic-based COVID-19 screening.

While the results of this study demonstrate the proposed system’s high classification accuracy and outline robustness considerations, the system is limited by the inherent flaws of crowdsourced databases. While some of these limitations were considered in this study with noise, recording device, and confounding factor analyses, some of the largest flaws in crowdsourced databases stems from issues of data reliability with subjects voluntarily reporting unverified information. This introduces the possibility of training models on incorrect labels. Furthermore, [66] suggests that using volunteer data for COVID detection causes limitations due to the different variants of COVID, which may cause symptoms to vary from subject to subject. Additional data with well-annotated and verified labels is required to fully understand the systems’ biases and address them.

The system performance was also found to degrade with simulated background noise, but added artificial noise is not a substitute for the dynamic interactions that real-world noise sources produce. Ideally, to address these concerns, training data would be collected that best matches the planned target deployment conditions. However, such an approach could limit the broad use of the system. Noise suppression techniques on the recordings (such as spectral subtraction, least means squared, etc.) or self-adaptation techniques to process the testing data to be more similar to the training data could achieve greater system performance and broad applicability.

Similar to other classification systems using neural networks, the predictions generated in this study lack interpretability and it is unclear what features contribute to the model’s classification decisions. Future work will include feature importance ranking to understand informative acoustic characteristics for COVID-19. Additionally, future work will include exploring the use of ensemble-based classification algorithms and adding training time as a performance metric to indicate the system’s low model complexity.

## 4. Conclusions

The global disruptions caused by COVID-19 have prompted research on improved test methods that can quickly and accurately identify the disease to prevent transmission. Acoustic-based detection systems offer the promise of scalable, rapid, and high-throughput screening tools. The proposed system demonstrates promising results as indicated by the performance in the Second DiCOVA Challenge where it obtained the highest average AUC-ROC of 0.83 when detecting COVID-19 subjects using breathing, cough, and speech sounds from the DiCOVA Blind Dataset. Additionally, the proposed system offers the promise of acoustic-based detection on spontaneous speech, which has the potential to allow for unobtrusive and continuous monitoring. However, it is clear that further work is needed in the field to offer clinical decision support and highly reliable diagnostics for at-home use.

This study highlights that considerations need to be made for noise contamination, variations in recording equipment, and the inherent variability of physiological sounds when deploying systems in real-world environments, challenges that have not been thoroughly examined to date. A framework for testing the robustness of a system is provided and preliminary results demonstrate pathways the authors plan to use to improve the system’s robustness. Importantly, results from acoustic-based COVID-19 classification can be further applied to acoustic classifications systems that have been proposed for other diseases such as asthma, tuberculosis, and pneumonia.

## Figures and Tables

**Table 1:** The percentages of recordings submitted with various confounding factors, including gender, age group, COVID symptoms, and other respiratory conditions.

	COVID	Gender			Age Group					COVID Symptoms			Other Respiratory		
													Condition		
	Positive	Female	Male	N/A	15–29	30–45	46–59	>60	N/A	Asymptomatic	Symptomatic	N/A	Yes	No	N/A
Breathing	17.0	23.4	69.5	7.1	14.7	11.3	3.8	0.7	69.5	4.2	24.3	71.5	2.2	28.9	68.8
Cough	17.1	23.4	69.7	6.9	14.7	11.3	3.8	0.7	69.5	4.2	24.3	71.5	2.2	29.1	68.7
Speech	19.7	27.5	72.5	0	15.9	12.7	4.2	1.9	65.3	3.5	15.4	81.1	1.4	27.3	71.3

**Table 2:** Feature dimensionality, classification model, and classification performance comparison to other proposed systems in the literature. All AUC-ROC scores reported for the systems compared were obtained when testing on the DiCOVA Blind Test set.

Reference	Sound Event	Classification Model	Complexity	AUC-ROC
Sharma et al. [22]	Cough	bidirectional Long Short-Term Memory	Moderate	0.75
Hoang et al. [60]	Cough	TRIpLet Loss Network based Light Gradient Boosting Machine	High	0.81
Ragolta et al. [61]	Cough	Contextual Attention based Convolutional Neural Network	High	0.68
Kamble et al. [62]	Cough	bidirectional Long Short-Term Memory	Moderate	0.77
*Proposed System*	Cough	Multilayer Perceptron	Low	0.79

## Data Availability

The data presented in this study are available at https://doi.org/10.48550/arXiv.2005.10548, https://github.com/drewgrant/COVIDAudioSocialMediaDataset and https://www.covid-19-sounds.org/en/blog/data_sharing.html (all accessed on 11 July 2022).
